# Supplementary material for: Vertebrate blood cell volume increases with temperature: implications for aerobic activity
Source: PeerJ. 2014 Apr 10;2:e346. doi: 10.7717/peerj.346 (PMC3994644; doi:10.7717/peerj.346)
Supplement: Appendix S1 — Data and sources used in Figs. 1 and 2. [file peerj-02-346-s001.docx]

**Appendix 1**: Data values for temperature (Temp.; ^o^ C), relative red blood cell volume (RRBC; % body mass), body mass (BM; g) and relative heart mass (RH; % body mass) used in analyses. Temperatures followed by one asterisk indicate that the temperature was estimated based on one or more species of the same genus. Temperatures followed by two asterisks indicate that the temperature estimate was based on the average temperature at which the species was held. Sources of data are listed in brackets.

| **Class** | **Order** | **Family** | **Species** | **Temp.** | |  |  | **RRBC** |  | | **BM** |  |  | **RH** |  |
| --- | --- | --- | --- | --- | --- | --- | --- | --- | --- | --- | --- | --- | --- | --- | --- |
| Aves | [Strigiformes](http://en.wikipedia.org/wiki/Owl) | Strigidae | *Bubo virginianus* | | 40.8 | [1] |  | 2 | [2] | 1495 | | [2] |  | 0.49 | [3] |
| Aves | [Falconiformes](http://en.wikipedia.org/wiki/Falconiformes) | [Accipitridae](http://en.wikipedia.org/wiki/Accipitridae) | *Buteo jamaicensis* | | 40.6 | [1] |  | 2.7 | [2] | 925 | | [2] |  | 0.55 | [3] |
| Aves | Columbiformes | Columbidae | *Columba livia* | | 40.8 | [4] |  | 4.9 | [2] | 310 | | [2] |  | 1 | [5] |
| Aves | Galliformes | [Phasianidae](http://en.wikipedia.org/wiki/Phasianidae) | *Coturnix japonica* | | 41.0 | [6] |  | 4.3 | [6] | 111 | | [6] |  | 0.88 | [5] |
| Aves | Struthioniformes | Dromaiidae | *Dromaius novaehollandiae* | | 38.0 | [1] |  | 3.7 | [7] | 40000 | | [7] |  | 0.85 | [8] |
| Aves | [Gruiformes](http://en.wikipedia.org/wiki/Gruiformes) | Rallidae | *Fulica americana* | | 40.9 | [1] |  | 4.4 | [2] | 550 | | [2] |  | 0.67 | [3] |
| Aves | [Charadriiformes](http://en.wikipedia.org/wiki/Charadriiformes) | Laridae | *Larus ridibundus******** | | 41.2 | [1] |  | 6.15 | [9] | 215 | | [9] |  | 0.95 | [9] |
| Aves | [Passeriformes](http://en.wikipedia.org/wiki/Passeriformes) | Sturnidae | *Sturnus vulgaris* | | 41.5 | [1] |  | 6.95 | [10] | 67 | | [10] |  | 1.32 | [5] |
| Aves | [Passeriformes](http://en.wikipedia.org/wiki/Passeriformes) | Emberizidae | *Zonotrichia querula* | | 42.5 | [1] |  | 3.78 | [11] | 38 | | [11] |  |  |  |
|  |  |  |  | |  |  |  |  |  |  | |  |  |  |  |
| Mammalia | Artiodactyla | Bovidae | *Bos taurus* | | 38.4 | [1] |  | 2.22 | [12] | 348000 | | [12] |  | 0.35 | [13] |
| Mammalia | Pilosa | [Bradypodidae](http://en.wikipedia.org/wiki/Bradypodidae) | *Bradypus tridactylus* | | 32.6 | [14] |  | 1.95 | [15] | 4133 | | [15] |  | 0.25 | [14] |
| Mammalia | Artiodactyla | Camelidae | *Camelus dromedarius* | | 37.2 | [1] |  | 2.4 | [16] | 310000 | | [16] |  |  |  |
| Mammalia | Artiodactyla | Bovidae | *Capra hircus* | | 39.5 | [1] |  | 2.04 | [17] | 40500 | | [17] |  | 0.5 | [18] |
| Mammalia | Rodentia | Caviidae | *Cavia porcellus* | | 39 | [1] |  | 3.23 | [19] | 500 | | [19] |  |  |  |
| Mammalia | [Perissodactyla](http://en.wikipedia.org/wiki/Odd-toed_ungulate) | Equidae | *Equus caballus* | | 37.4 | [20] |  | 2.85 | [21] | 415000 | | [21] |  | 0.82 | [13] |
| Mammalia | Primates | Cercopithecidae | *Macaca mulatta* | | 39.3 | [1] |  | 2.96 | [22] | 4400 | | [22] |  | 0.50 | [5] |
| Mammalia | Rodentia | Cricetidae | *Mesocricetus auratus* | | 38.1 | [1] |  | 2.72 | [23] | 94 | | [23] |  | 0.92 | [5] |
| Mammalia | Rodentia | Muridae | *Mus musculus* | | 36.7 | [1] |  | 3.16 | [24] | 24 | | [24] |  |  |  |
| Mammalia | Chiroptera | Vespertilionidae | *Myotis lucifugus* | | 37.2 | [1] |  | 6.5 | [25] | 9 | | [1] |  | 1.03 | [26] |
| Mammalia | Lagomorpha | [Leporidae](http://en.wikipedia.org/wiki/Leporidae) | *Oryctolagus cuniculus* | | 39 | [1] |  | 2.56 | [27] | 3900 | | [27] |  | 0.29 | [13] |
| Mammalia | Artiodactyla | Cervidae | *Rangifer tarandus* | | 39.2 | [1] |  | 3.46 | [28] | 90300 | | [28] |  | 0.71 | [5] |
| Mammalia | Rodentia | Muridae | *Rattus norvegicus* | | 37.2 | [1] |  | 2.24 | [29] | 222 | | [29] |  | 0.55 | [30] |
| Mammalia | Diprotodontia | [Macropodidae](http://en.wikipedia.org/wiki/Macropodidae) | *Setonix brachyurus* | | 36.3 | [1] |  | 2.47 | [31] | 3250 | | [31] |  |  |  |
| Mammalia | Artiodactyla | Suidae | *Sus scrofa* | | 38.6 | [32] |  | 3.1 | [33] | 147000 | | [33] |  |  |  |
| Mammalia | [Diprotodontia](http://en.wikipedia.org/wiki/Diprotodontia) | [Phalangeridae](http://en.wikipedia.org/wiki/Phalangeridae) | *Trichosurus vulpecula* | | 36 | [1] |  | 2.62 | [34] | 2020 | | [34] |  | 0.37 | [35] |
|  |  |  |  | |  |  |  |  |  |  | |  |  |  |  |
| Reptilia | [Crocodylia](http://en.wikipedia.org/wiki/Crocodylia) | [Alligatoridae](http://en.wikipedia.org/wiki/Alligatoridae) | *Alligator mississippiensis* | | 29.9 | [36] |  | 1.4 | [37] | 2304 | | [37] |  | 0.15 | [38] |
| Reptilia | Squamata | Agamidae | *Amphibolurus caudicinctus* | | 36* | [39] |  | 3.1 | [40] | 23 | | [40] |  |  |  |
| Reptilia | Squamata | Agamidae | *Amphibolurus inermis* | | 36* | [39] |  | 3.7 | [40] | 24 | | [40] |  |  |  |
| Reptilia | Squamata | Agamidae | *Amphibolurus ornatus* | | 36* | [39] |  | 2.2 | [40] | 23 | | [40] |  |  |  |
| Reptilia | Squamata | [Boidae](http://en.wikipedia.org/wiki/Boidae) | *Boa constrictor* | | 30.7 | [41] |  | 1.9 | [37] | 1715 | | [37] |  | 0.21 | [5] |
| Reptilia | [Crocodylia](http://en.wikipedia.org/wiki/Crocodylia) | [Crocodylidae](http://en.wikipedia.org/wiki/Crocodylidae) | *Crocodylus moreleti* | | 31.5* | [42] |  | 1.4 | [37] | 2058 | | [37] |  |  |  |
| Reptilia | [Crocodylia](http://en.wikipedia.org/wiki/Crocodylia) | [Crocodylidae](http://en.wikipedia.org/wiki/Crocodylidae) | *Crocodylus niloticus* | | 31.5 | [42] |  | 1.3 | [37] | 3500 | | [37] |  | 0.12 | [5] |
| Reptilia | Squamata | Viperidae | *Crotalus viridis* | | 28.9 | [43] |  | 1.29 | [44] | 372 | | [44] |  | 0.25 | [45] |
| Reptilia | Squamata | Colubridae | *Elaphe obsoleta* | | 27.8 | [39] |  | 1.07 | [44] | 541 | | [44] |  | 0.17 | [45] |
| Reptilia | Testudines | Testudinidae | *Gopherus polyphemus* | | 34.5 | [43] |  | 1.9 | [37] | 4114 | | [37] |  |  |  |
| Reptilia | Squamata | Iguanidae | *Iguana iguana* | | 28.1 | [46] |  | 1.8 | [37] | 465 | | [37] |  | 0.19 | [47] |
| Reptilia | Squamata | [Elapidae](http://en.wikipedia.org/wiki/Elapidae) | *Laticauda colubrina* | | 29 | [48] |  | 1.93 | [49] | 417 | | [49] |  |  |  |
| Reptilia | Squamata | Colubridae | *Pituophis catenifer* | | 26.7 | [43] |  | 1.9 | [37] | 672 | | [37] |  |  |  |
| Reptilia | Squamata | Iguanidae | *Sauromalus obesus* | | 37.9 | [43] |  | 2.85 | [50] | 200 | | [50] |  |  |  |
|  |  |  |  | |  |  |  |  |  |  | |  |  |  |  |
| Amphibia | Anura | Bufonidae | *Bufo marinus* | | 25.2 | [51] |  | 1.93 | [52] | 200 | | [52] |  | 0.6 | [5] |
| Amphibia | Anura | Bufonidae | *Bufo melanostictus* | | 29 | [53] |  | 3.4 | [54] | 55 | | [54] |  |  |  |
| Amphibia | Anura | Hylidae | *Hyla septentrionalis* | | 24.7 | [55] |  | 1.7 | [55] | 32 | | [55] |  |  |  |
| Amphibia | Anura | Ranidae | *Lithobates catesbeiana* | | 21.3* | [51] |  | 1.28 | [56] | 344 | | [56] |  | 0.23 | [5] |
| Amphibia | Anura | Ranidae | *Rana esculenta* | | 28 | [57] |  | 2.06 | [58] | 20 | | [58] |  | 0.28 | [5] |
| Amphibia | Anura | Ranidae | *Rana pipiens* | | 24.4 | [51] |  | 1.84 | [59] | 50 | | [59] |  | 0.33 | [60] |
|  |  |  |  | |  |  |  |  |  |  | |  |  |  |  |
| [Actinopterygii](http://en.wikipedia.org/wiki/Actinopterygii) | [Acipenseriformes](http://en.wikipedia.org/wiki/Acipenseriformes) | [Acipenseridae](http://en.wikipedia.org/wiki/Acipenseridae) | *Acipenser fulvescens* | | 16 | [61] |  | 0.9 | [62] | 3058 | | [62] |  |  |  |
| [Actinopterygii](http://en.wikipedia.org/wiki/Actinopterygii) | [Amiiformes](http://en.wikipedia.org/wiki/Amiiformes) | Amiidae | *Amia calva* | | 30.5 | [63] |  | 1.2 | [62] | 1963 | | [62] |  |  |  |
| [Actinopterygii](http://en.wikipedia.org/wiki/Actinopterygii) | [Anguilliformes](http://en.wikipedia.org/wiki/Anguilliformes) | Anguillidae | *Anguilla japonica* | | 18** | [64] |  | 1.04 | [64] | 199 | | [64] |  |  |  |
| [Actinopterygii](http://en.wikipedia.org/wiki/Actinopterygii) | [Semionotiformes](http://en.wikipedia.org/wiki/Semionotiformes) | [Lepisosteidae](http://en.wikipedia.org/wiki/Lepisosteidae) | *Atractosteus tristoechus* | | 22.5** | [65] |  | 1 | [65] | 1628 | | [65] |  |  |  |
| [Actinopterygii](http://en.wikipedia.org/wiki/Actinopterygii) | [Cypriniformes](http://en.wikipedia.org/wiki/Cypriniformes) | [Catostomidae](http://en.wikipedia.org/wiki/Catostomidae) | *Catostomus commersoni* | | 24 | [66] |  | 1 | [62] | 617 | | [62] |  |  |  |
| [Actinopterygii](http://en.wikipedia.org/wiki/Actinopterygii) | [Cypriniformes](http://en.wikipedia.org/wiki/Cypriniformes) | [Cyprinidae](http://en.wikipedia.org/wiki/Cyprinidae) | *Cyprinus carpio* | | 25** | [67] |  | 1.44 | [67] | 529 | | [67] |  | 0.16 | [68] |
| [Actinopterygii](http://en.wikipedia.org/wiki/Actinopterygii) | [Perciformes](http://en.wikipedia.org/wiki/Perciformes) | [Serranidae](http://en.wikipedia.org/wiki/Serranidae) | *Epinephelus striatus* | | 25 | [69] |  | 0.8 | [62] | 1270 | | [62] |  |  |  |
| [Actinopterygii](http://en.wikipedia.org/wiki/Actinopterygii) | [Gadiformes](http://en.wikipedia.org/wiki/Gadiformes) | [Gadidae](http://en.wikipedia.org/wiki/Gadidae) | *Gadus morhua* | | 7** | [70] |  | 0.6 | [70] | 4200 | | [70] |  | 0.12 | [71] |
| [Actinopterygii](http://en.wikipedia.org/wiki/Actinopterygii) | [Cypriniformes](http://en.wikipedia.org/wiki/Cypriniformes) | [Catostomidae](http://en.wikipedia.org/wiki/Catostomidae) | *Ictiobus cyprinellus* | | 22 | [72] |  | 0.9 | [62] | 3395 | | [62] |  |  |  |
| [Actinopterygii](http://en.wikipedia.org/wiki/Actinopterygii) | [Perciformes](http://en.wikipedia.org/wiki/Perciformes) | [Lutjanidae](http://en.wikipedia.org/wiki/Lutjanidae) | *Lutjanus campechanus* | | 24.7 | [73] |  | 0.9 | [62] | 3765 | | [62] |  |  |  |
| [Actinopterygii](http://en.wikipedia.org/wiki/Actinopterygii) | [Perciformes](http://en.wikipedia.org/wiki/Perciformes) | [Lutjanidae](http://en.wikipedia.org/wiki/Lutjanidae) | *Lutjanus griseus* | | 27.0 | [74] |  | 0.7 | [62] | 3711 | | [62] |  |  |  |
| [Actinopterygii](http://en.wikipedia.org/wiki/Actinopterygii) | [Perciformes](http://en.wikipedia.org/wiki/Perciformes) | [Serranidae](http://en.wikipedia.org/wiki/Serranidae) | *Mycteroperca tigris* | | 28 | [74] |  | 1 | [62] | 5885 | | [62] |  |  |  |
| [Actinopterygii](http://en.wikipedia.org/wiki/Actinopterygii) | [Salmoniformes](http://en.wikipedia.org/wiki/Salmoniformes) | Salmonidae | *Oncorhynchus mykiss* | | 16** | [75] |  | 0.67 | [75] | 815 | | [75] |  | 0.11 | [76] |
| [Actinopterygii](http://en.wikipedia.org/wiki/Actinopterygii) | [Perciformes](http://en.wikipedia.org/wiki/Perciformes) | Sparidae | *Pagrus major* | | 19.5** | [67] |  | 1.27 | [67] | 519 | | [67] |  |  |  |
| [Actinopterygii](http://en.wikipedia.org/wiki/Actinopterygii) | [Acipenseriformes](http://en.wikipedia.org/wiki/Acipenseriformes) | [Polyodontidae](http://en.wikipedia.org/wiki/Paddlefish) | *Polyodon spathula* | | 22 | [77] |  | 0.8 | [62] | 4679 | | [62] |  | 0.13 | [78] |
| [Actinopterygii](http://en.wikipedia.org/wiki/Actinopterygii) | [Perciformes](http://en.wikipedia.org/wiki/Perciformes) | Scaridae | *Pseudoscarus guacamaia* | | 28 | [74] |  | 1.2 | [62] | 4607 | | [62] |  |  |  |
| [Actinopterygii](http://en.wikipedia.org/wiki/Actinopterygii) | [Perciformes](http://en.wikipedia.org/wiki/Perciformes) | [Carangidae](http://en.wikipedia.org/wiki/Carangidae) | *Seriola quinqueradiata* | | 21.4** | [67] |  | 1.23 | [67] | 873 | | [67] |  |  |  |
| [Actinopterygii](http://en.wikipedia.org/wiki/Actinopterygii) | [Perciformes](http://en.wikipedia.org/wiki/Perciformes) | [Carangidae](http://en.wikipedia.org/wiki/Carangidae) | *Seriola quinqueradiata* | | 23.7** | [79] |  | 1.2 | [79] | 433 | | [79] |  |  |  |
| [Actinopterygii](http://en.wikipedia.org/wiki/Actinopterygii) | [Perciformes](http://en.wikipedia.org/wiki/Perciformes) | [Sphyraenidae](http://en.wikipedia.org/wiki/Sphyraenidae) | *Sphyraena barracuda* | | 24.4 | [80] |  | 0.9 | [62] | 2204 | | [62] |  |  |  |
| [Actinopterygii](http://en.wikipedia.org/wiki/Actinopterygii) | [Perciformes](http://en.wikipedia.org/wiki/Perciformes) | [Scombridae](http://en.wikipedia.org/wiki/Scombridae) | *Thunnus albacares* | | 25** | [82] |  | 1.38 | [82] | 1071 | | [82] |  | 0.29 | [81] |

REFERENCES

1. Clarke A, Rothery P. 2008. Scaling of body temperature in mammals and birds. *Functional Ecology* 22(1):58-67.

2. Bond CF, Gilbert CW. 1958. Comparative study of blood volume in representative aquatic and nonaquatic birds. *American Journal of Physiology* 194(3): 519-521.

3. Hartman FA. 1955. Heart weight in birds. *The Condor* 57(4): 221-238.

4. Østnes JA, Bech C. 1998. Thermal control of metabolic cold defence in pigeons, *Columba livia*. *Journal of Experimental Biology* 201: 793-803.

5. Vinogradov AE, Anatskaya OV. 2006. Genome size and metabolic intensity in tetrapods: a tale of two lines. *Proceedings of the Royal Society B* 273: 27-32.

6. Weathers WW, Snyder GK. 1973. Functional acclimation of Japanese quail to simulated high altitude. *Journal of Comparative Physiology* 93: 127-137.

# 7. Dawson TJ, Herd RM, Skadhauge E. 1983. Water turnover and body water distribution during dehydration in a large arid-zone bird, the Emu, Dromaius novaehollandiae. *Journal of Comparative Physiology*, 153(2): 235-240.

8. Grubb B, Jorgensen DD, Conner M. 1983. Cardiovascular changes in the exercising emu. *Journal of Experimental Biology*, 104: 193-201.

9. Viscor G, Marqués MS, Palomeque J. 1985. Cardiovascular and organ weight adaptations as related to flight activity in birds. *Comparative Biochemistry and Physiology Part A: Physiology* 82(3): 597-599.

10. Palacios L, Palomeque J, Riera M, Pages T, Viscor G, Planas J. 1984. Oxygen transport properties in the starling, *Sturnus vulgaris*. *Comparative Biochemistry and Physiology* 77A (2): 255-260.

# 11. deGraw, WA, Kern MD. 1985. Changes in the blood and plasma volume of Harris sparrows during postnuptial molt. *Comparative Biochemistry and Physiology*, 81A (4):889-893.

12. Chaiyabutr N, Preuksagorn S, Komolvanich S, Chanpongsang S. 2000. Comparative study on the regulation of body fluids and mammary circulation at different stages of lactation in crossbred Holstein cattle feeding on different types of roughage. *Journal of Animal Physiology and Animal Nutrition* 83: 74-84.

13. Crile G, Quiring DP. 1940. A record of the body weight and certain organ and gland weights of 3690 animals. *The Ohio Journal of Science* 40: 219-285.

14. Oliveira LHA, Da Costa CP, Huggins SE. 1980. Cardiac mass, blood temperature and ventricular fibrillation: A study of the comparative physiology of the three-toed sloth and the domestic cat. *Comparative Biochemistry and Physiology Part A: Physiology* 67(A): 483-490.

15. Bozzini CE, Henriques JAP, Ladosky W. 1978. Characterization of the blood volume and iron kinetics in the three-toed sloth, *Bradypus tridactylus*. *Comparative Biochemistry and Physiology Part A: Physiology* 61(3): 417-418.

16. Banerjee S, Bhattacharjee RC. 1963. Distribution of body water in the camel (*Camelus dromedarius*) *American Journal of Physiology* 204(6): 1045-1047.

17. McKean T, Walker B. 1974. Comparison of selected cardiopulmonary parameters between the pronghorn and the goat. *Respiration Physiology* 21(3): 365-370.

18. Jürgens KD, Bartels H, Bartels R. 1981. Blood oxygen transport and organ weights of small bats and small non-flying mammals. *Respiration Physiology* 45(3): 243-260.

19. Ancill RJ. 1956. The blood volume of the normal guinea-pig. *Journal of Physiology* 132: 469-475.

20. Mortola JP, Lanthier C. 2004. Scaling the amplitudes of the circadian pattern of resting oxygen consumption, body temperature and heart rate in mammals. *Comparative Biochemistry and Physiology, Part A* 139: 83-95.

21. Carlson GP. 1987. Hematology and body fluids in the equine athlete: a review. In *Equine Exercise Physiology* (eds. Gillespie JR, Robinson NE, Davis CA), pp. 393-424. Davis, CA, ICEEP Publications.

# 22. Forsyth RP, Hoffbrand BI, Melmon KI. 1970. Redistribution of cardiac output during hemorrhage in the unanesthetized monkey. *Circulation Research,* 27:311-320.

23. Fitts DA, Corp ES, Simpson JB. 1982. Salt appetite and intravascular volume depletion following colloid dialysis in hamsters. *Behavioral and Neural Biology* 34: 75-88.

24. Riches AC, Sharp JG, Thomas DB, Smith SV. 1973. Blood volume determination in the mouse. *Journal of Physiology* 228: 279-284.

25. Kallen, FC. 1960. Plasma and blood volumes in the little brown bat. *American Journal of Physiology*, 198(5): 999-1005.

26. Armstrong RB, Lanuzzo CD, Kunz TH. 1977. [Histochemical and biochemical properties of flight muscle fibers in the little brown bat, *Myotis lucifugus*](http://link.springer.com/article/10.1007/BF00686562). *Journal of Comparative Physiology*, 119:141-154.

27. Tsutsumi Y, Oguri N. 1972. Blood volume changes in pregnant and pseudopregnant rabbits. *Journal of the Faculty of Agriculture, Hokkaido University* 57(1): 1-24.

28. Cameron RD, Luick JR. 1972. Seasonal changes in total body water, extracellular fluid, and blood volume in grazing reindeer. *Canadian Journal of Zoology* 50(1): 107-116.

29. Lee HB, Blaufox MD. 1985. Blood volume in the rat. *Journal of Nuclear Medicine* 25: 72-76.

30. Martin RR, Haynes H. 1970. Application of LaPlace's law to mammalian hearts. *Comparative Biochemistry and Physiology* 34: 959-962.

31. Shield J. 1971. A seasonal change in blood cell volume of the Rottnest Island Quokka, *Setonix brachyurus*. *Journal of Zoology* 165(3): 343-354.

32. Hannon JP, Bassone CA, Wade CE. 1989. Normal physiological values for conscious pigs used in biomedical research. *Division of Military Trauma Research*, Institute Report 89: 1-15.

33. Anderson DM, Elsley FWH, McDonald I. 1970. Blood volume changes during pregnancy and lactation of sows. *Journal of Experimental Physiology*, 55:293-300.

34. Dawson TJ, Denny MJS. 1968. Influence of the spleen on blood volume and haematocrit in the brush-tailed possum (*Trichosurus vulpecula*). *Australian Journal of Zoology* 16(4): 603-608.

35. Gilmore DP. 1984. Organ-body weight relationships in the common brushtail possum *Trichosurus vulpecula*. *Australian Journal of Mammalogy* 7: 131-138.

36. Seebacher F, Elsey RM, 3rd. TP. 2003. Body temperature null distributions in reptiles with nonzero heat capacity: seasonal thermoregulation in the American alligator (*Alligator mississippiensis*). *Physiological and Biochemical Zoology* 76(3): 348-359.

37. Thorson TB. 1968. Body fluid partitioning in reptilia. *Copeia* 3: 592-601.

38. Coulson RD, Herbert JD, Coulson TD. 1989. Biochemistry and physiology of alligator metabolism in vivo. *American Zoologist* 29(3): 921-934.

39. Clusella-Trullas S, Blackburn TM, Chown SL. 2011. Climatic predictors of temperature performance curve parameters in ectothermy imply complex responses to climate change. *American Naturalist* 177(6): 738-751.

40. Bradshaw SD. 1970. Seasonal changes in the water and electrolyte metabolism of Amphibolurus lizards in the field. *Comparative Biochemistry and Physiology* 36(4): 689-717.

41. McGinnis SM, Moore RG. 1969. Thermoregulation in the Boa constrictor. *Herpetologica* 25(1): 38-45.

42. Downs CT, Greaver C, Taylor R. 2008. Body temperature and basking behaviour of Nile crocodiles (*Crocodylus niloticus*) during winter. *Journal of Thermal Biology* 33: 185-192.

43. Brattstrom BH. 1965. Body temperatures of reptiles. *American Midland Naturalist* 73(2): 376-422.

44. Lillywhite HB, Smits AW. 1984. Lability of blood volume in snakes and its relation to activity and hypertension. *Journal of Experimental Biology* 110: 267-274.

45. Seymour RS. 1987. Scaling of cardiovascular physiology in snakes. *American Zoologist* 27(1): 97-109.

46. Tosini G, Menaker M. 1995. Circadian rhythm of body temperature in an ectotherm (*Iguana iguana*). *Journal of Biological Rhythms* 10(3): 285-295.

47. Franz RG, Hummel J, Kienzle E, Kölle P, Gunga H-C, Clauss M. 2009. Allometry of visceral organs in living amniotes and its implications for sauropod dinosaurs. *Proceedings of the Royal Society B* 276(1662): 1731-1736.

48. Heatwole H, Grech A, Monahan JF, King S, Mars H. 2012. Thermal biology of sea snakes and sea kraits. *Integrative and Comparative Biology* 52(2): 257-273.

49. Pough FH, Lillywhite HB. 1984. Blood volume and blood oxygen capacity of sea snakes. *Physiological Zoology* 57(1): 32-39.

50. Nagy KA. 1972. Water and electrolyte budgets of a free-living desert lizard, *Sauromalus obesus*. *Journal of Comparative Physiology* 79(1): 39-62.

51. Brattstrom BH. 1963. A preliminary review of the thermal requirements of amphibians. *Ecology* 44(2): 238-255.

52. Baustian M. 1988. The contribution of lymphatic pathways during recovery from hemorrage in the toad, *Bufo marinus*. *Physiological Zoology* 61(6): 555-563.

53. Boral MC, Deb CC. 1970. Seasonal changes in body fluids and haematology in toad *Bufo melanostictus* a poikilothermic cold torpor. *Proceedings of the Indian National Science Academy* 36(6): 369-379.

54. Boral MC, Deb CC. 1970. Seasonal changes in body fluids and haematology in toad *Bufo melanostictus* a poikilothermic cold torpor. *Proceedings of the Indian National Science Academy* 36(6), 369-373.

55. Carmena-Suero A, Siret JR, Caixejas J, Arpones-Carmena D. 1980. Blood volume in male *Hyla* *septentrionalis* (tree frog) and *Rana catesbeiana* (bullfrog). *Comparative Biochemistry and Physiology Part A: Physiology* 67(1): 187-189.

56. Hillman SS, DeGrauw EA, Hoagland T, Hancock T, Withers P. 2010. The role of vascular and interstitial compliance and vascular volume in the regulation of blood volume in two species of anuran. *Physiological and Biochemical Zoology* 83(1): 55-67.

57. Hofer R, Ladurner H, Gattringer A, Wieser W. 1975. Relationship between the temperature preferenda of fishes, amphibians and reptiles, and the substrate affinities of their trypsins. *Journal of Comparative Physiology* 99(4): 345-355.

58. Frangioni G, Borgioli G. 1994. Hepatic respiratory compensation and blood volume in the frog (*Rana esculenta*). *Journal of Zoology, Lond*on 234: 601-611.

59. Rouf MA. 1969. Hematology of the leopard frog, *Rana pipiens*. *Copeia* 4: 682-687.

60. Smith VDE, Jackson CM. 1931. The changes during dessication and rehydration in the body and organs of the leopard frog (*Rana pipiens*). *The Biological Bulletin* 60(1): 80-93.

61. Patrick HK, Sutton TM, Swink WD. 2007. Application of a dichotomous key to the classification of sea lamprey *Petromyzon marinus* marks on lake sturgeon *Acipenser fulvescens.* *Great Lakes Fisheries Commission Special Publication* 2: 1-25.

62. Thorson TB. 1961. The partitioning of body water in Osteichthyes: phylogenetic and ecological implications in aquatic vertebrates. *Biological Bulletin* 120(2): 238-254.

63. Reynolds WW, Casterlin ME, Millington ST. 1978. Circadian rhythm of preferred temperature in the bowfin *Amia calva*, a primitive holostean fish. *Comparative Biochemistry and Physiology Part A: Physiology* 60(1): 107-109.

64. Takei Y. 1988 Changes in blood volume after alteration of hydromineral balance in conscious eels, *Anguilla japonica*. *Comparative Biochemistry and Physiology* 91A (2): 293-297.

65. Siret JA, Carmena AO, Callejas J. 1976. Erythrokinetic study in the fish manjuari (*Atractosteus tristoechus*). *Comparative Biochemistry and Physiol*ogy 55A: 127-128.

66. Jobling M. 1981. Temperature tolerance and the final preferendum-rapid methods for the assessment of optimum growth temperatures. *Journal of Fish Biology* 19: 439-455.

67. Itazawa Y, Takeda T, Yamamoto K, Azuma T. 1983. Determination of circulating blood volume in three teleosts, carp, yellowtail and porgy. *Japanese Journal of Ichthyology* 30(1): 94-101.

68. Bass A, Ošťádal B, Pelouch V, Vítek V. 1973. Differences in weight parameters, myosin-ATPase activity and the enzyme pattern of energy supplying metabolism between the compact and spongious cardiac musculature of carp (*Cyprinus carpio*) and turtle (*Testudo horsfieldi*). *Pflügers Archiv* 343(1): 65-77.

69. Colin PL. 1992. Reproduction of the Nassau grouper, *Epinephelus striatus* (Pisces: Serranidae) and its relationship to environmental conditions. *Environmental Biology of Fishes* 34: 357-377.

70. Ronald K, Macnab HC, Stewart JE, Beaton B. 1964. Blood properties of aquatic vertebrates: I. Total blood volume of the Atlantic cod, *Gadus morhua* L. *Canadian Journal of Zoology* 42(6): 1127-1132.

71. Mendonca PC, Genge AG, Deitch EJ, Gamperl AK. 2007 Mechanisms responsible for the enhanced pumping capacity of the in situ winter flounder heart (*Pseudopleuronectes* *americanus*). *American Journal of Physiology* 293: R2112–R2119.

72. Wismer DA., Christie AE. 1987. Temperature relationships of great lakes fishes: A data compilation. *Great Lakes Fisheries Commission Special Publication* 87(3): 165.

73. Rivas LR. 1970. Snappers of the Western Atlantic. *Commercial Fisheries Review* 32(1): 41.

74. Garcia CB, Duarte LO. 2002. Consumption to biomass (Q/B) ratio and estimates of Q/B-predictor parameters for Caribbean fishes. *Naga, The ICLARM Quarterly* 25(2): 19-31.

75. Nikinmaa M, Soivio A, Railo E. 1981. Blood volume of *Salmo gairdneri*: Influence of ambient temperature. *Comparative Biochemistry and Physiology* 69A: 767-769.

76. Farrell AP, Hammons AM, Graham MS, Tibbits GF. 1988. Cardiac growth in rainbow trout, *Salmo gairdneri*. *Canadian Journal of Zoology* 66: 2368-2373.

77. Crance JH. 1987. Habitat suitability index curves for paddlefish, developed by the Delphi technique. *North American Journal of Fisheries Management* 7(1): 123-130.

78. Simeanu C, Simeanu D, Nistor C, Pasarin B. 2011. Evaluation of quantitative particularities of meat production at *Polyodon spathula* species. *Lucrari Stiintifice* 56: 312-315.

79. Yamamoto K, Itazawa Y, Kobayashi H. 1979. Supply of erythrocytes into the circulating blood from the spleen of exercised fish. *Comparative Biochemistry and Physiology* 65A: 5-11.

80. O’Toole AC, Danylchuk AJ, Suski CD, Cooke SJ. 2010. Consequences of catch-and-release angling on the physiological status, injury, and immediate mortality of great barracuda (*Sphyraena barracuda*) in the Bahamas. *ICES Journal of Marine Science* 67: 1667–1675.

81. Farrell AP, Davie PS, Franklin CE, Johansen JA, Brill RW. 1992. Cardiac physiology in tunas. I. In vitro perfused heart preparations from yellowfin and skipjack tunas. *Canadian Journal of Zoology* 70: 1200-1210.

82. Brill R, Cousins K, Jones DR, Bushness PG, Steffensen JF. 1998. Blood volume, plasma volume and circulation time in a high-energy-demand teleost, the yellowfin tuna (*Thunnus albacares*). *Journal of Experimental Biology* 201: 647-654.
